# Preparation and cutting performance study of YSZ-toughened PcBN superhard tools

**DOI:** 10.1039/d3ra02079g

**Published:** 2023-05-23

**Authors:** Yuxiao Yue, Yumei Zhu, Zhihong Li

**Affiliations:** a Key Laboratory for Advanced Ceramics and Machining Technology of Ministry of Education, School of Materials Science and Engineering, Tianjin University Tianjin 300072 China tjuzhuyumei@163.com +86 022-27404260 +86 022-27404260

## Abstract

Titanium alloy, as a recognized difficult-to-cut material, places higher demands on the performance of cutting tools. Compared with the mainstream cemented carbide tools, PcBN tools have a higher life and better machining performance. In this paper, a new type of cubic boron nitride superhard tool was prepared by introducing Y_2_O_3_-stabilized ZrO_2_ (YSZ) under high temperature and high pressure (1500 °C, 5.5 GPa), and the effect of the variation of YSZ addition on the mechanical properties of the tool was systematically analyzed, and the cutting performance of the tool was also analyzed by cutting TC4. It was found that a small amount of YSZ addition, which generated a sub-stable t-ZrO_2_ phase during the sintering process, could improve the mechanical properties of the tool and increase its cutting life. When YSZ was added at 5 wt%, the flexural strength and fracture toughness of the composites reached the maximum values of 637.77 MPa and 7.18 MPa m^1/2^, while the cutting life of the tools reached the maximum value of 2615.81 m. And when YSZ was added at 2.5 wt%, the hardness of the material reached the maximum value of 43.62 GPa.

## Abstract 2

Titanium alloy, as a recognized difficult-to-cut material, places higher demands on the performance of cutting tools.

## Graphical abstract

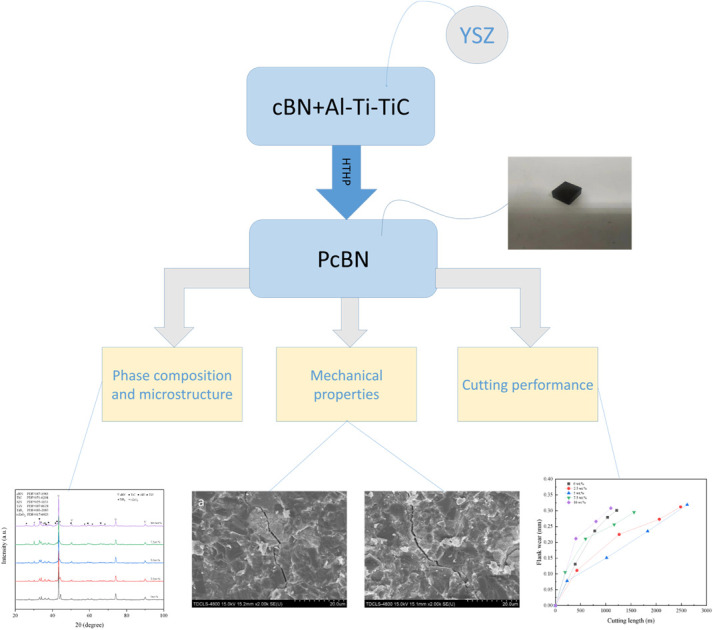

## 1. Introduction

Titanium alloys have high strength, good toughness and superior high temperature properties, and are widely used in aerospace, automotive manufacturing and military applications. Titanium alloys are commonly used in the manufacture of aircraft engine pressurized components, and are also important structural components for rockets, missiles, and other aircraft.^1–3^ Titanium alloys have good corrosion resistance and fatigue resistance, good biocompatibility, and have also gained wide popularity in the biomedical field. Titanium alloys are now commonly used to manufacture medical surgical instruments, such as scalpels, and surgical forceps. Also, titanium alloys are used to make artificial joint materials and orthodontic devices.^4^

However, titanium alloy is recognized as a difficult-to-cut material, and in the process of cutting and machining, there are a series of problems such as small deformation coefficient, high cutting temperature, high cutting force per unit area, and serious machining hardening phenomenon. For the cutting and machining of titanium alloy, the mainstream tools are currently carbide tools, such as YG6, YD15, *etc.* When machining titanium alloy, carbide tools face problems such as short machining life, low surface accuracy, and when the cutting speed of the tool exceeds 45 m min^−1^, it will lead to a large amount of diffusion and loss of tool elements.^5,6^

With the emergence and development of high-speed cutting lathes and superhard cutting tools, high-speed cutting has gradually become the mainstream machining method for titanium alloys.^7,8^ High-speed cutting technology has the advantages of high efficiency, high precision and high surface quality. Due to the low thermal conductivity of titanium alloys, the heat generated under high-speed cutting causes a rapid increase in the temperature at the cutting place of the tool, resulting in rapid damage to the tool kerf, fragmentation and the formation of work hardening.^9–11^ Therefore, high speed cutting places higher demands on the toughness, thermal stability and life of the tool. Polycrystalline cubic boron nitride (PcBN) tools are a new type of superhard tools with excellent cutting characteristics such as high thermal stability and good chemical stability, excellent thermal conductivity, low coefficient of friction and high hardness second only to diamond tools, all of which are beneficial for high-speed cutting of titanium alloys.^12–17^ In recent years, research on the cutting performance of PcBN tools for titanium alloys has been progressing, and how to prepare high-performance PcBN tools has become a major hot issue for research. Mo Peicheng, Chen Chao *et al.* prepared PcBN composite inserts with Al–Ti–W as binder at 1550 °C and 5.5 GPa, and found that when the W content was 6 wt%, the inserts had optimal mechanical properties, with hardness and flexural strength reaching 30.71 GPa and 972.3 MPa, respectively.^18^ Eriki, Ananda Kumar *et al.* investigated the cutting performance of PcBN on titanium alloy (gr2) and obtained smooth cutting surfaces at cutting speeds of 15–45 m min^−1^, feeds of 0.1–0.2 mm/*r*, and back draft of 0.1 mm.^19^ Liu Zhanqiang *et al.* studied the tool wear morphology and wear mechanism of PcBN high-speed cutting of titanium alloy TC4, and proved that bonded wear, diffuse wear and brittle wear are the main wear mechanisms when PcBN high-speed cutting titanium alloy.^20^ In this study, new PcBN tools were prepared using Al, Ti and TiC as binding agents and YSZ as reinforcing phase, and the effect of YSZ addition on the mechanical properties and cutting performance of PcBN tools was investigated. YSZ generates sub-stable t-ZrO_2_ during high temperature sintering and recooling, and undergoes phase transformation from t-ZrO_2_ to m-ZrO_2_ when subjected to stress, which inhibits the extension of material cracks and improve the performance of PcBN tools.

## 2. Experimental procedures

### 2.1. Zirconia phase transition toughening

Zirconia exists in three crystalline forms: monoclinic zirconia (m-ZrO_2_), tetragonal zirconia (t-ZrO_2_) and cubic zirconia (c-ZrO_2_). The phase transition from m-ZrO_2_ to t-ZrO_2_ occurs when the temperature is increased to 1170 °C. When the temperature is cooled down to 950 °C, t-ZrO_2_ is retransformed to m-ZrO_2_, and this process is accompanied by a 5% volume expansion effect. By adding appropriate amount of stabilizers such as MgO, Y_2_O_3_, *etc.* to ZrO_2_, the phase transformation process of tetragonal zirconia during cooling can be inhibited, which makes the material matrix disperse sub-stable t-ZrO_2_ at room temperature, and when the applied load is applied, the sub-stable t-ZrO_2_ will undergo the phase transformation process of t → m by stress to produce volume expansion, which inhibits the expansion of cracks within the material matrix under the action of external forces, so that the strength and toughness of the material can be improved.^21–23^

Zhao, Du *et al.*^24^ analyzed the toughening mechanism of ZrO_2_-toughened Al_2_O_3_ ceramics and pointed out that the conversion rate of t-ZrO_2_ to m-ZrO_2_ is the main factor affecting the ceramic composites. Li, Zhang *et al.*^25^ prepared ZrO_2_-doped ZrB_2_–MoSi_2_ composites, and the doping of zirconia led to a significant improvement in the mechanical properties of the material with a 50% increase in toughness. Jiang Wentao *et al.*^26^ improved both the fracture toughness and hardness of WC-Co cemented carbide materials by introducing ZrO_2_ into the material, in which the fracture toughness of the carbide was significantly improved, which was related to the microcrack deflection caused by the phase transformation of t-ZrO_2_ inside the material under stress-induced effects. Zang, Han *et al.*^27,28^ investigated the effect of ZrO_2_ introduction on the mechanical properties of PcBN and PCD materials and improved the fracture toughness by introducing Y_2_O_3_ into the materials to stabilize ZrO_2_, and pointed out that the toughening of ZrO_2_ is mainly in the form of stress-induced phase change toughening and microcrack toughening.

### 2.2. Sample preparation

The raw materials include: cBN powders (particle size: 2.5–20 μm; purity >99%), Al powders (particle size: 1–2 μm; purity >99%), Ti powders (particle size: less than 28 μm; purity >99%), TiC powders (particle size: 1–2 μm; purity >99%), YSZ powders (particle size: 0.5–1 μm; Y_2_O_3_ content = 8%). The content of the bonding agent Al–Ti–TiC in the composites was 16 wt%, and the content of the additive YSZ was 0, 2.5 wt%, 5.0 wt%, 7.5 wt%, and 10 wt%, respectively.

Firstly, the powder raw materials were put into the planetary ball mill for wet ball milling, the grinding medium was ZrO_2_ balls, the mass ratio of powder, balls and ethanol was 1 : 2 : 2, the ball milling speed was 400 rpm, and the ball milling time was 3 h. After the completion of ball milling, the mixed raw materials were dried in a vacuum drying oven for 48 h, and the ethanol was allowed to evaporate completely. The powders were then compacted in a graphite mold and reduced under vacuum at 380 °C for 24 h to eliminate gas adsorption and possible oxidation on the surface of the binder powder. The mixed powder was then placed in a 6 × 8 MN hexahedral top press for sintering (temperature: 1500 °C; pressure: 5.5 GPa; holding time: 90 s).

**Fig. 1:**
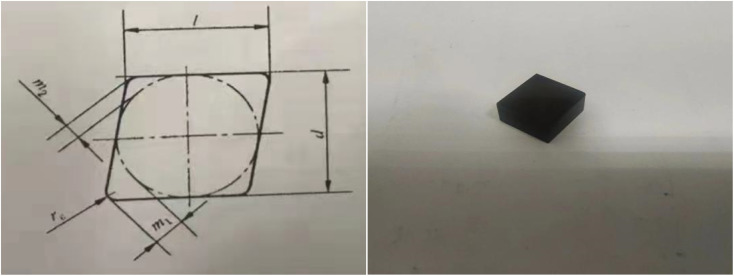
PcBN tool machining diagram and finished product appearance diagram.

The sintered block is machined into a suitable size PcBN tool by machining the size code CN N120408S, and the specific size of the tool is shown in Table 1, unit: mm. The machining dimensions of the tool and the shape of the finished product are shown in Fig. 1.

**Table 1:** PcBN tool processing size

*d*	*l*	*m*_1_	*m*_2_	*s*	*α*_n_	*γ*_ε_
12.7	12.9	3.088	1.697	4.76	0°	0.8

### 2.3. Characterization

The phase composition of the samples was examined using X-ray diffractometer (XRD, MiniFlex600, Rigaku, Japan). The sample structure was characterized using a field emission electron scanning microscope (FESEM, s-4800, Hitachi, Japan). The composition and distribution of the sample elements were characterized using an energy spectrometer (EDS, x-max20, Oxford, Britain). The bending strength of the samples was determined by the three-point bending method on a universal testing machine (XWW, Beijing Jinshengxin detecting instrument Co., Ltd, China). The relative density was determined using the Archimedes drainage method. The Vickers hardness and toughness of the polished surface of the specimens were determined using Vickers hardness tester (HMAS-010, Shanghai Runguang Tech., China). The thermal stability of fractured samples was tested by the differential thermogravimetric analyzer (NETZSCH449C, NETZSCH, Germany).The cutting test of TC4 was performed on a CNC lathe (CK6136, Guangzhou Numerical Control Equipment Co. Ltd, GuangZhou, China) with the prepared PcBN composite insert (*v*_c_ = 130 m min^−1^, *v*_f_ = 0.1 mm/*r*, *α*_p_ = 0.1 mm). The surface machining accuracy of the workpiece was measured by surface roughness meter.

The degree of wear on the flank surface of the tool was measured using an optical microscope and the PcBN tool was identified as having failed when the average wear on the flank surface of cutting tool reached 0.3 mm.^29^ The tool life of PCBN was characterized by counting the total cutting length at the time of tool failure.

## 3. Result and discussion

### 3.1. Phase composition and microstructure

**Fig. 2:**
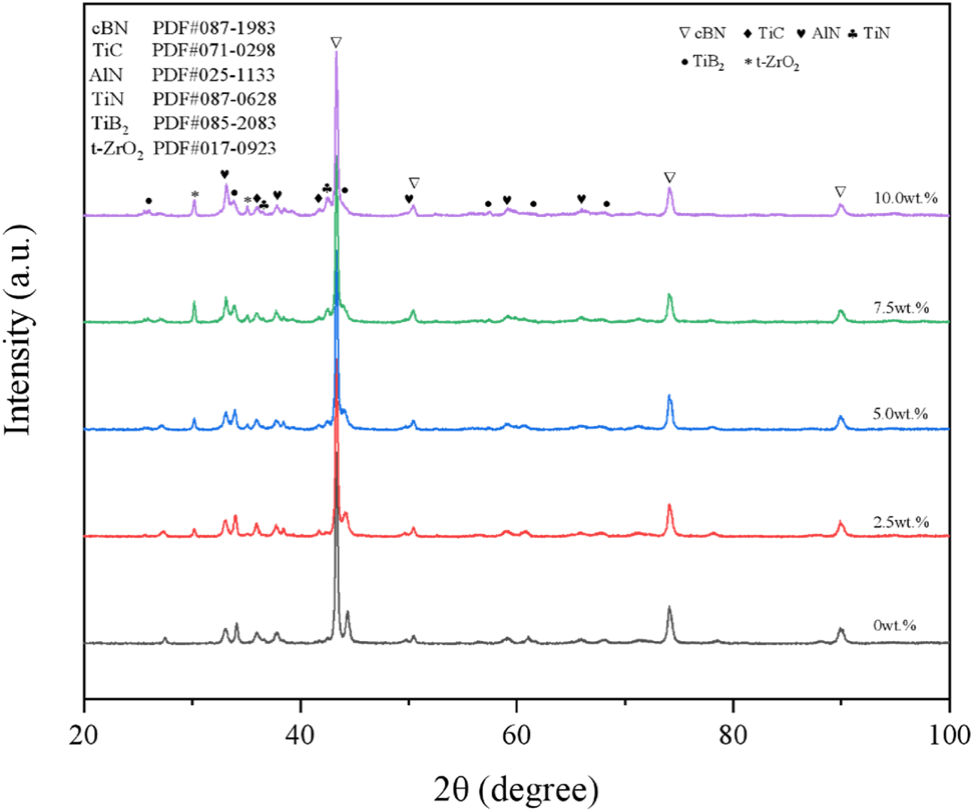
XRD patterns of samples with different YSZ content.

Fig. 2 shows the XRD spectra of the materials with different YSZ additions. From the figure, it can be seen that the composition of the material phase includes cBN, TiC, AlN and TiB_2_ when YSZ is not introduced into the material system, and the diffraction peaks of Al and Ti are not observed in the spectra, which shows that during the sintering process, the metal phase will bond with the matrix cBN particles by reaction under high temperature and pressure, and AlN, TiN and TiB_2_ are generated.^12,18^ TiC itself undergoes slow oxidation in air at high temperatures, but the diffraction peaks of TiO_2_ are not observed in the phase diagram, while no diffraction peaks of other carbides appear, indicating that TiC does not oxidize or react with other components of the material system during the sintering process, and is dispersed inside the material body in the form of particles. During the sintering process, factors such as higher pressure and shorter sintering time avoid the occurrence of TiC oxidation.

With the addition of YSZ, the diffraction peak of t-ZrO_2_ appeared at 36° in the XRD spectrum, and the intensity increased with the addition of YSZ, which proved that the transformation from monoclinic to tetragonal crystalline type was completed by YSZ at high temperature and high pressure. The diffraction peak of m-ZrO_2_ was not found in the spectrum, which indicates that the phase transformation process of t-ZrO_2_ to m-ZrO_2_ at low temperature was suppressed under the condition of Y_2_O_3_ stabilization, and the sub-stable t-ZrO_2_ phase was obtained.^27,30^

**Fig. 3:**
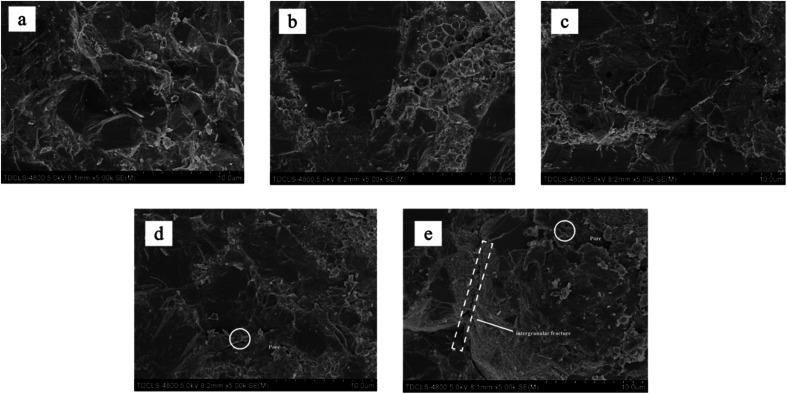
SEM images of samples microstructure: (a) 0 wt%, (b) 2.5 wt%, (c) 5 wt%, (d) 7.5 wt%, (e) 10 wt%.

**Fig. 4:**
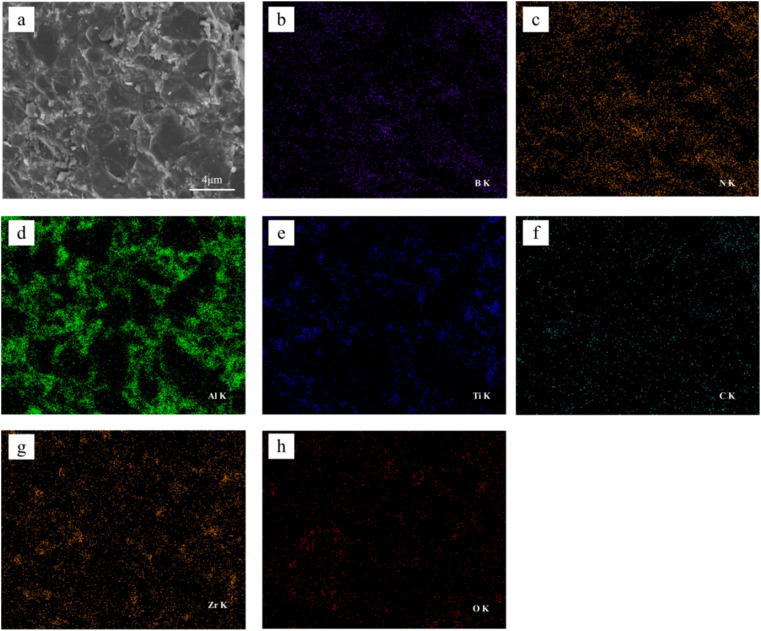
SEM image and corresponding EDS maps of the sample with 5 wt% YSZ.

Fig. 3 shows the SEM images of the fracture morphology of the samples, from which we can see that when the YSZ addition is low, a denser PCBN composite can be obtained by high temperature and high pressure sintering. At the YSZ addition higher than 7.5 wt%, pores appeared inside the material (Fig. 3d and e), which caused the degradation of the material properties. As seen in Fig. 3e, when YSZ was added at 10 wt%, significant porosity and cracks were observed inside the material body. The surface elements of the samples with 5 wt% YSZ addition were analyzed using EDS, and the distribution of each element is shown in Fig. 4. Comparing Fig. 4b and c diagrams, it can be seen that B and N elements have similar distribution, mainly in the form of BN, which is distributed on the grain surface. Al and Ti are mainly distributed at grain boundaries (Fig. 4d and e), and Al and Ti melt into liquid phase under high temperature conditions, and the flowing mass transfer of molten metal occurs within the system, causing the rearrangement of CBN particles to achieve The closest arrangement promotes the sintering densification. Combined with the previous XRD phase diagram, it can be seen that the molten Al and Ti fill in the grain gap of cBN particles and react with cBN particles to form AlN, TiN and TiB_2_, which firmly hold the cBN particles and enhance the bonding of the composite. The relatively uniform distribution of Zr in the composites indicates that ZrO_2_ does not participate in the reaction during the sintering process, but is mainly distributed on the surface and around the cBN particles in the form of a second phase.

### 3.2. Mechanical properties

**Fig. 5:**
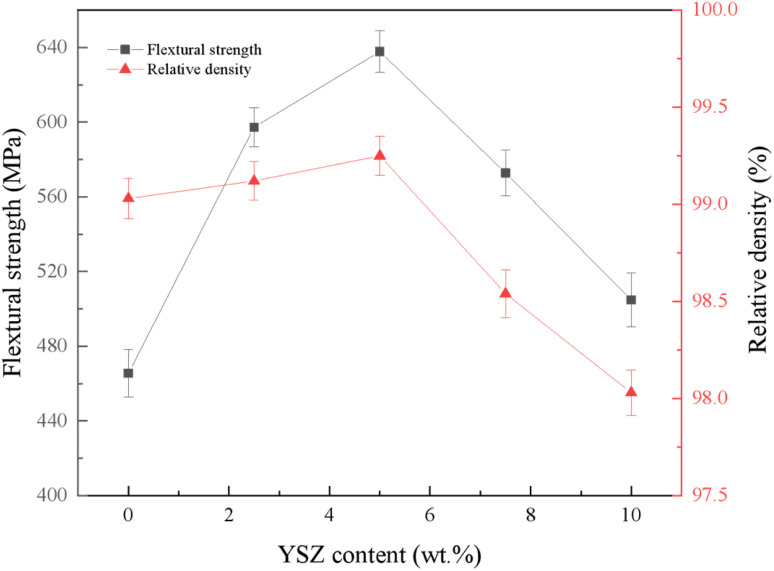
Flexural strength and relative density of samples with different YSZ content.

Fig. 5 shows the effect of YSZ addition on the relative density and flexural strength of the composites. As can be seen from the figure, the relative density changes first increase and then decrease with the increase of YSZ addition. The addition of appropriate amount of YSZ can improve the dense material because YSZ particles effectively fill the interfacial voids of CBN particles, which provides good interfacial bonding for the PcBN matrix and increases the bonding of the composite. When YSZ was added at 5 wt%, the relative density of the composite reached a maximum of 99.25%, which was 4.2% higher than that without YSZ. The addition of excessive YSZ increases the difficulty of dispersion and leads to agglomeration of particles, causing a series of negative effects such as decrease in material density and increase in porosity, which in turn leads to a decrease in the mechanical properties of the material. The flexural strength of the samples showed a trend of increasing and then decreasing with the addition of YSZ, and the flexural strength of the samples reached 637.77 MPa when YSZ was added at 5 wt%, which was 37.0% higher than that without YSZ. The strengthening effect of YSZ on the material can be summarized as follows: first, the introduction of ZrO_2_ particles in the sample in the right amount can play the role of dispersion strengthening; second, ZrO_2_ itself has high strength and high toughness, and its introduction in the right amount can significantly improve the mechanical properties of the material; third, when cracks occur inside the material, the t-ZrO_2_ around the cracks will undergo a phase change process to m-ZrO_2_ by stress, absorbing the energy of crack expansion, while volume expansion occurs to produce compressive stress on the cracks and hinder the further expansion of the cracks.^22,31^

**Fig. 6:**
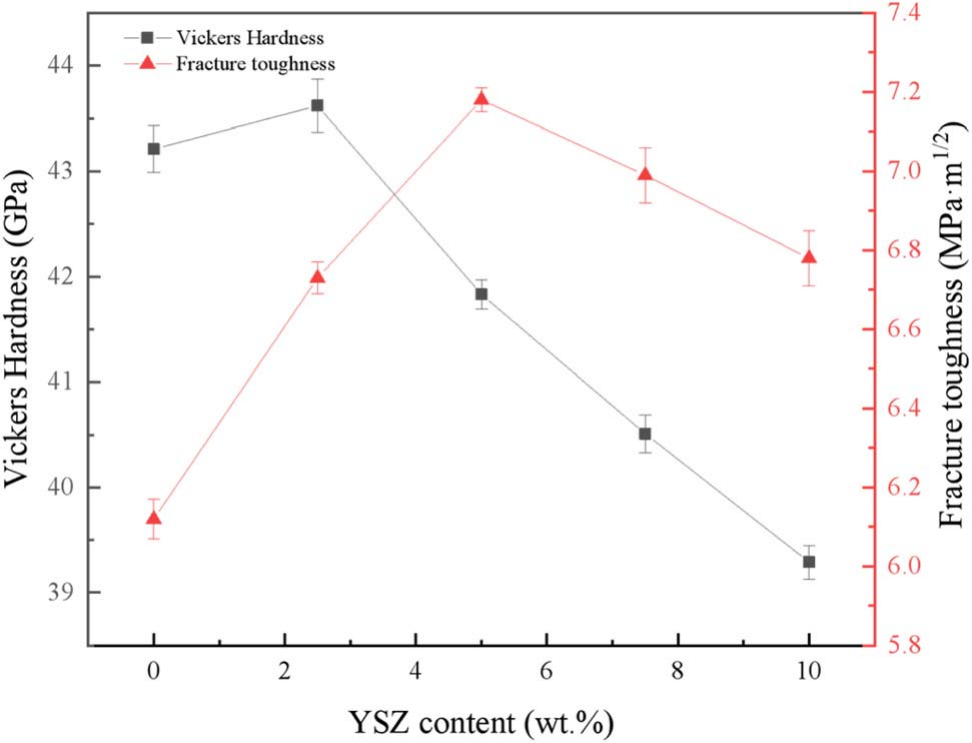
Vickers Hardness and fracture toughness of samples with different YSZ content.

Fig. 6 reflects the variation of Vickers hardness and fracture toughness of the material with the addition of YSZ. At 2.5 wt% YSZ addition, the hardness of the composite reached a maximum value of 43.62 GPa, which is 9.5% higher than that without YSZ addition. The increase in hardness was mainly attributed to the sintering densification promoted by the addition of small amount of YSZ. With the increase of YSZ addition, the hardness of the material decreased because the hardness of the second phase YSZ is lower than that of the matrix cBN, and the increase of the added content leads to the decrease of the hardness of the composite. In addition, the addition of excess YSZ causes agglomeration within the material, resulting in increased porosity within the material, which affects the sinterability of the composite, and this is also one of the reasons for the decrease in hardness. The fracture toughness of the material reflects the trend of increasing and then decreasing with the increase of YSZ addition, and the fracture toughness of the material can be significantly improved by introducing an appropriate amount of ZrO_2_ into the material. The fracture toughness of the material reached 7.18 MPa m^1/2^ at a YSZ addition of 5 wt%, which was 17.3% higher than that of the sample without YSZ addition.

**Fig. 7:**
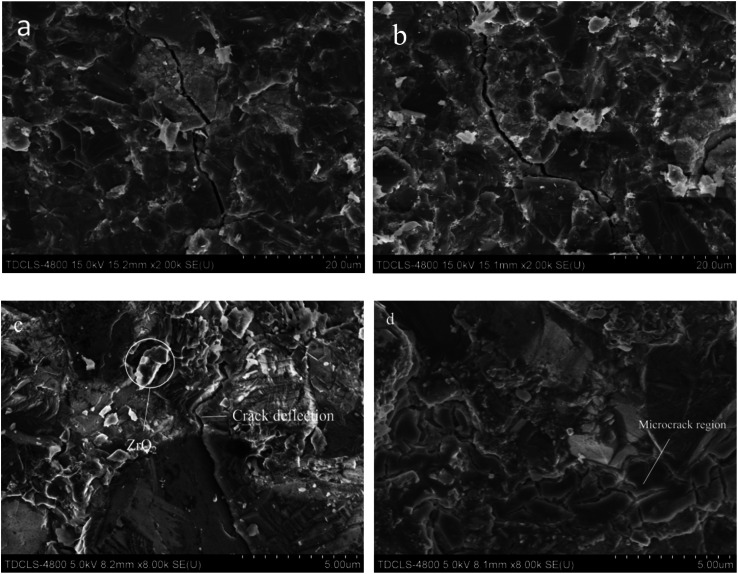
SEM images of appearance of the sample fracture: (a) 0 wt%, (b) 5 wt%, (c) 5 wt%, (d) 5 wt%.

**Fig. 8:**
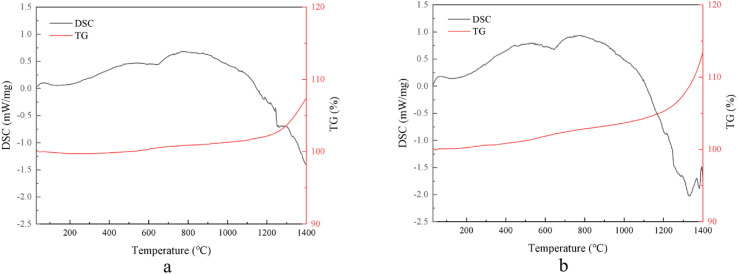
Differential thermal curves of fracture samples with different YSZ content: (a) 0 wt%, (b) 5 wt%.

Fig. 7 shows the crack expansion of the material at 0 wt% and 5 wt% YSZ addition. The toughening of materials by ZrO_2_ is mainly achieved through stress-induced phase change toughening and microcrack toughening.^27,32^ From Fig. 7c, it can be seen that during the crack extension, the ZrO_2_ particles near the crack are subjected to the stress transformation of t-ZrO_2_ → m-ZrO_2_ and accompanied by 5% volume expansion, and the increase in volume causes compressive stress on the crack and causes the deflection of the crack. To further confirm the occurrence of zirconia phase transition in the material, the fractured PcBN specimens were selected for differential thermal analysis (see Fig. 8), and it can be seen that the DSC curve of PcBN with 5 wt% YSZ added shows a heat-absorbing phase transition peak at 1300 °C compared to the specimen without YSZ, which represents the phase transition from m-ZrO_2_ to t-ZrO_2_, indicating the material body the presence of internal m-ZrO_2_ phase, which is produced by the transformation of sub-stable t-ZrO_2_ inside the specimen body by stress during fracture.^33^ Meanwhile, the presence of microcracks inside the material body can be observed in Fig. 7d, which is also produced by the volume effect occurring in ZrO_2_ during the phase transformation. When the material fractures, the main crack extends to the microcrack region and needs to cross the microcrack to achieve further extension, which requires more energy consumption, and the generation of the microcrack region plays a blunt role in the extension of the main crack. Therefore, the fracture toughness of the material can be significantly improved by introducing an appropriate amount of ZrO_2_ into the PcBN composite.

### 3.3. Cutting performance

**Fig. 9:**
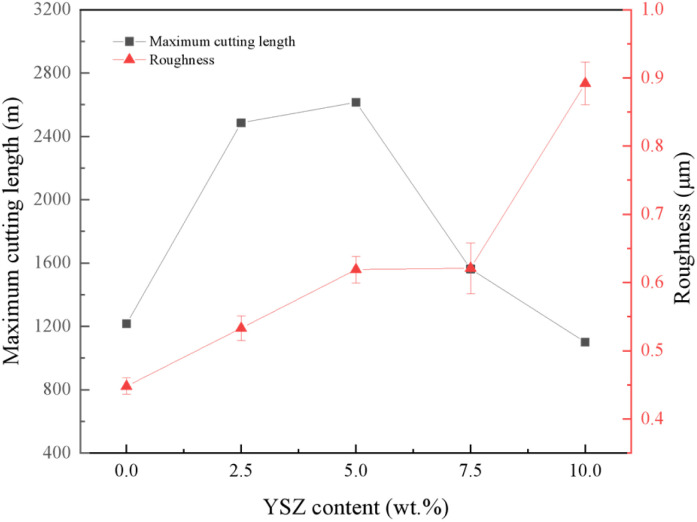
Maximum cutting length and workpiece roughness of samples with different YSZ content.

Fig. 9 reflects the tool life and machining accuracy of the tools with different YSZ additions. The tool life increases and then decreases with the increase of YSZ additions for TC4 cutting by PcBN tools. At 5 wt% YSZ addition, the maximum cutting length of the tool reached 2615.81 m, which was 215.04% higher than that of the tool without YSZ addition. The maximum cutting length of the material showed a similar variation pattern as the fracture toughness, which can be deduced that the increase of fracture toughness is beneficial to the improvement of cutting life of the tool when machining titanium alloy. The introduction of high strength and high toughness ZrO_2_ into the PcBN matrix can reduce the wear of the tool surface and extend the tool life when cutting TC4. The surface roughness of TC4 increased with the addition of YSZ increased from 0.440 μm to 0.868 μm. At low YSZ addition, the surface roughness of the workpiece was in the range of 0.45–0.65 μm and increased slightly with YSZ addition. Excessive YSZ addition leads to a significant decrease in the surface roughness of the workpiece, which may be caused by the poor sintering bonding of the material due to the inhomogeneous dispersion of ZrO_2_ in the interior and the tendency of particle shedding during the cutting process of TC4.

**Fig. 10:**
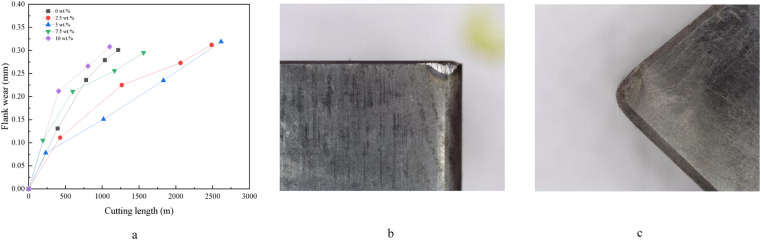
Tool wear curves for different YSZ additions and the wear morphology of tool with 5 wt% YSZ content.

Fig. 10 a reflects the variation of the degree of wear on its back tool face with increasing cutting length for PcBN tools with different YSZ additions. From the figure, it can be seen that the wear rate of several tools is larger at the beginning of cutting, and gradually decreases with the increase of the wear level. Combined with the wear morphology of the rear tool face (Fig. 10b), it can be seen that the wear of PcBN tools is dominated by the adhesive wear of the rear tool face. During high speed cutting of TC4, the heat generated in cutting is concentrated at the cutting edge due to the low thermal conductivity, which makes the temperature at the machining position rise rapidly. The higher temperature makes the titanium alloy have high chemical activity and affinity, and the high cutting temperature and small cutting area between the tool and the workpiece provide an ideal environment for the diffusion of elements between the two. The adhering material causes changes in the geometry of the tool, resulting in tool wear. Some small crescent-shaped chips can be observed at the edges of the front tool face, and it can be inferred that PcBN tools cutting titanium alloys are also subject to brittle breakage such as chipping and flaking under the effect of mechanical and thermal impacts.^20,34–36^

## 4. Conclusions

1. The dense PcBN composites were prepared by high temperature and high pressure method. After introducing YSZ into the material, the added ZrO_2_ was uniformly dispersed inside the material mainly as a tetragonal phase, and Al reacted with Ti during the sintering process to generate AlN, TiN and TiB_2_, which promoted the bonding between the cBN particles.

2. By introducing YSZ, the mechanical properties of the composites could be effectively improved. The hardness of the composites increased to 43.62 GPa when YSZ was added at 2.5 wt%. The flexural strength and fracture toughness of the composites reached the maximum values of 637.77 MPa and 7.18 MPa m^1/2^ when YSZ was added at 5%. The improvement of the mechanical properties of the materials was the result of the synergistic effect of dispersive particle strengthening, zirconia phase change toughening and microcrack toughening.

3. Compared to the PcBN tool without YSZ addition, the maximum cutting length of the PcBN tool reached 2615.81 m at 5 wt% addition, an increase of 215.04%. The increase in tool life was mainly attributed to the increase in material toughness. The machined surface roughness of TC4 gradually increased with the addition of YSZ. Therefore, the cutting life of the tool can be improved by adding the reinforcing phase ZrO_2_ to the preparation of PcBN composite inserts, as far as the surface accuracy of the workpiece allows.

## Conflicts of interest

We declare that we do not have any commercial or associative interest that represents a conflict of interest in connection with the work submitted.

## Supplementary Material
